# Skin transcriptome profiling of Changthangi goats highlights the relevance of genes involved in Pashmina production

**DOI:** 10.1038/s41598-020-63023-6

**Published:** 2020-04-08

**Authors:** Sonika Ahlawat, Reena Arora, Rekha Sharma, Upasna Sharma, Mandeep Kaur, Ashish Kumar, Karan Veer Singh, Manoj Kumar Singh, Ramesh Kumar Vijh

**Affiliations:** 1 ICAR-National Bureau of Animal Genetic Resources, Karnal, India; 2 ICAR-Central Institute for Research on Goats, Mathura, India

**Keywords:** Biotechnology, Molecular biology

## Abstract

Pashmina, the world’s finest natural fiber is derived from secondary hair follicles of Changthangi goats which are domesticated in Ladakh region of Jammu and Kashmir by nomadic pastoralists. Complex epithelial-mesenchymal interactions involving numerous signal molecules and signaling pathways govern hair follicle morphogenesis and mitosis across different species. The present study involved transcriptome profiling of skin from fiber type Changthangi goats and meat type Barbari goats to unravel gene networks and metabolic pathways that might contribute to Pashmina development. In Changthangi goats, 525 genes were expressed at significantly higher levels and 54 at significantly lower levels with fold change >2 (p_adj_ < 0.05). Functional annotation and enrichment analysis identified significantly enriched pathways to be formation of the cornified envelope, keratinization and developmental biology. Expression of genes for keratins (KRTs) and keratin-associated proteins (KRTAPs) was observed to be much higher in Changthangi goats. A host of transcriptional regulator genes for hair follicle keratin synthesis such as GPRC5D, PADI3, HOXC13, FOXN1, LEF1 and ELF5 showed higher transcript abundance in Pashmina producing goats. Positive regulation of Wnt signaling pathway and negative regulation of Oncostatin M signaling pathway may be speculated to be important contributors to hair follicle development and hair shaft differentiation in Changthangi goats.

## Introduction

Thirty four registered breeds and many non-descript populations represent the diverse caprine genetic resources of India which inhabit four eco-zones viz. Temperate Himalayan, North Western, Southern Peninsular and Eastern region. There is great variability in Indian goats with regard to many phenotypic traits such as color, size, reproductive and productive parameters. Additionally, on the basis of utility, they are classified into milk, meat and fiber type breeds (www.nbagr.res.in). An important breed of extremely cold Temperate Himalayan region of India is Changthangi which is a source of world’s most luxurious natural fiber, Pashmina/Cashmere. Hence, these goats are also referred to as Pashmina or Cashmere goats. The ability of this breed to adapt to high altitude hypoxic conditions has enabled it to thrive in difficult terrains of Changthang region of Ladakh and hilly tract of Leh. In contrast, Barbari is a breed of North Western region, which is primarily reared for milk and meat purpose. These animals are small sized, short-haired and white coloured with small light brown patches^1^.

Pashmina is considered the world’s finest natural fiber and is obtained from undercoat of goats native to Asia. Indian Pashmina is mainly derived from Changthangi goats which are domesticated in Ladakh region of Jammu and Kashmir by nomadic pastoralists (*Changpas*) and is a prominent symbol of their cultural heritage. It is also known as Cashmere, Kashmir and pashm in India (http://jksheephusbandrykashmir.nic.in). The other Cashmere producing countries are China, Mongolia, Iran, Afghanistan, Pakistan and Nepal. Owing to its warmth, fineness, acclaimed aesthetic value and lusture, it has rightly been called “soft gold” or “the king of fibers”. The premium price it fetches is understandable in light of its greater softness than even superfine merino of the same diameter^2^. Cashmere is derived from secondary hair follicles of goats which undergo cyclic variation as a result of complex epithelial-mesenchymal interactions. These physiological processes involve interactions of numerous signal molecules and signaling pathways which govern hair follicle morphogenesis and mitosis^3^.

Transcriptomic profiling using the Illumina high-throughput sequencing platform has opened new vistas for unraveling of global gene expression and annotation of whole transcriptomes underpinning phenotypic and physiological variability. Such advances in genome research have facilitated an improved understanding of systemic gene expression and ensuing regulatory mechanisms in several species^4^. Over the last five years, Cashmere goats have become focus of intense study and there have been attempts to identify molecular mechanisms governing hair follicle morphogenesis^5^, hair follicle cycling under natural and shortened photoperiod conditions^4^, and also, delineating the gene networks controlling the coat color in these goats^6^. However, to the best our knowledge, there are no reports on comparative transcriptome profiling of skin of goats inhabiting contrasting climatic regions and differing in terms of utility, particularly from the Indian subcontinent. Therefore, the present study was planned to elucidate complete repertoire of transcripts expressed in the skin of fiber type Changthangi goats from cold desert region of India and compare it with meat type Barbari goats from hot arid regions of the country. Novel information from the caprine skin transcriptomes can contribute to elucidation of genetic networks determining adaptation to divergent agro-ecological zones and differences in the quality of the fiber produced by the two breeds under study.

## Results

In order to quantify the gene expression patterns of goat skin samples, cDNA libraries were constructed from 4 animals each of Changthangi and Barbari breeds and these libraries were subjected to deep sequencing using Illumina HiSeq platform.

### Summary of RNA Seq data

The number of raw reads and processed reads varied from 42.1 to 55.5 million and 40.8 to 53.8 million, respectively for different samples. Mapping with the *Capra hircus* reference assembly ARS1 yielded 93.24 to 95.85% aligned reads, suggesting good quality of RNA-seq data for further analysis. Similarity in the percentage of mapped reads eliminated any sequencing biases in the dataset generated. Detailed results are presented in Table 1.

**Table 1:** Summary of read statistics of 8 libraries from Barbari and Changthangi breeds.

Sample details	Number of raw reads	Processed reads	Percent of high quality data	Percent aligned reads	Number of genes expressed
Barbari1	42,180,760	40,871,412	96.90	94.33	17,891
Barbari2	44,027,632	42,757,303	97.11	94.41	18,089
Barbari3	43,653,637	42,326,495	96.96	93.24	17,923
Barbari4	46,000,800	44,554,407	96.86	94.22	17,870
Changthangi1	52,572,185	50,994,026	97.00	95.85	18,756
Changthangi2	51,395,618	49,913,541	97.12	94.35	17,721
Changthangi3	55,521,228	53,856,077	97.00	95.14	18,461
Changthangi4	53,981,415	51,928,597	96.20	94.42	19,521

### Functional enrichment analysis

**Figure 1:**
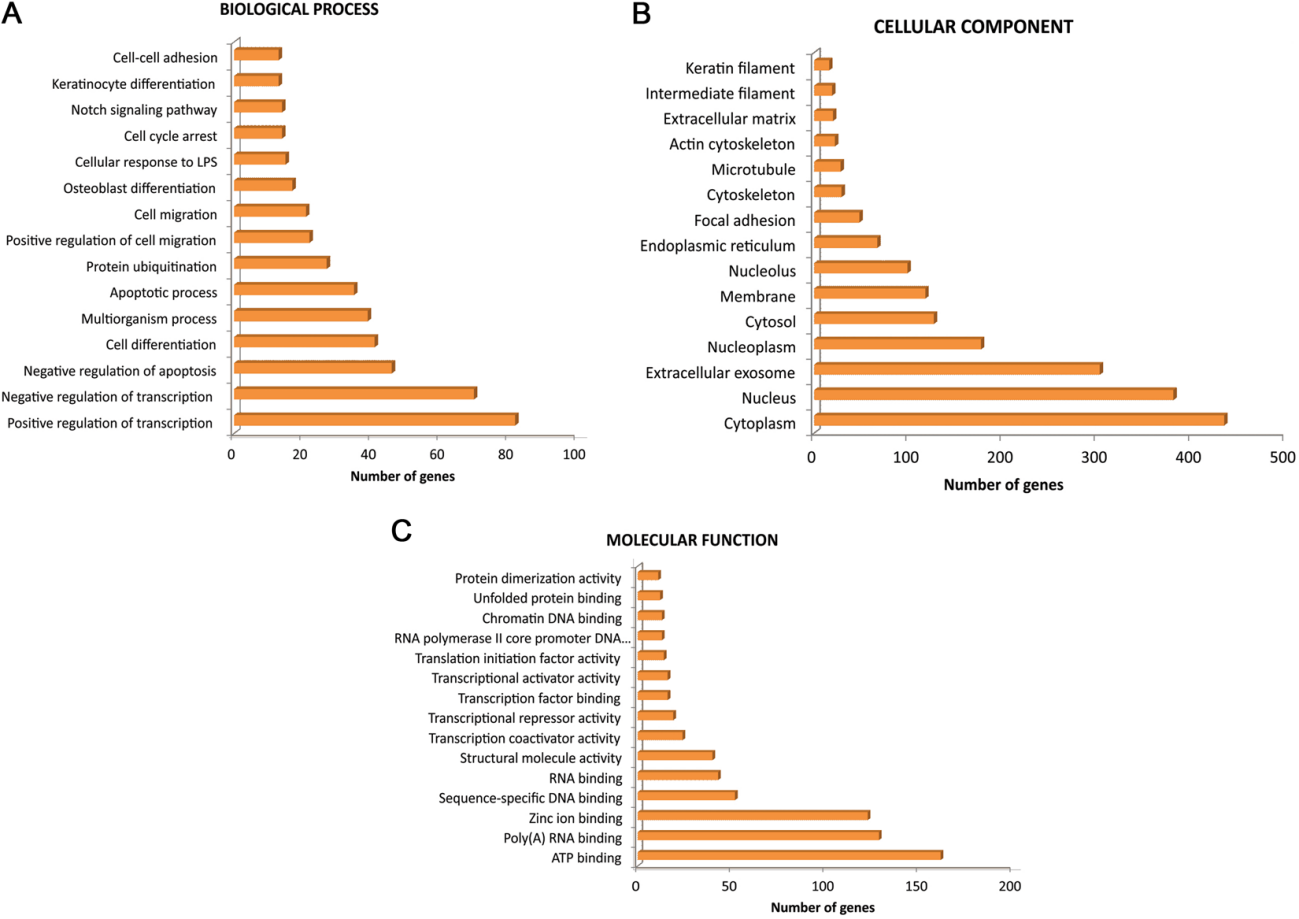
Gene Ontology terms for differentially expressed genes between Chanthangi and Barbari goats (2095 genes, p_adj_ < 0.05) ((**A**) Biological Process; (**B**) Cellular Component and (**C**) Molecular Function).

Based on annotation of the *Bos taurus* genome, the top 30 genes with highest expression in both breeds were linked to biological functions such as cellular macromolecule metabolic process, cellular biosynthetic process, skin development, intermediate filament cytoskeleton organization, hair follicle morphogenesis and epidermis morphogenesis. A total of 1147 genes were expressed at significantly higher levels and 948 at significantly lower levels in Changthangi goats (p_adj_ < 0.05) as compared to Barbari goats. Gene Ontology enrichment analysis was performed for the differentially expressed genes (DEGs). Top 5 enriched functional categories included signal, coiled coil, intermediate filament, cytoskeleton, keratin and differentiation. Classification of the DEGs into biological process (BP), cellular components (CC) and molecular function (MF) is detailed in Fig. 1.

On the basis of KEGG enrichment analysis, the DEGs were observed to be involved in several pathways which include protein processing in endoplasmic reticulum, transcriptional misregulation in cancer, ribosome biogenesis in eukaryotes, adherens junction, FoxO signaling pathway, AMPK signaling pathway and extracellular matrix receptor interaction. Genes related to adherens junction were ACTB, PTPRJ, CREBBP, CSNK2B, LEF1, SMAD3, CTNND1, ACTN1, NECTIN4, TCF7L1, VCL, IGF1R, SORBS1, FYN and SSX2IP. Genes representing extracellular matrix receptor interaction pathway included ITGA1, HSPG2, VWF, ITGA9, CD47, LAMC3, LAMA5, ITGB7, COMP, ITGB6, SV2B, THBS1, COL11A1, FN1 and SPP1.

**Figure 2:**
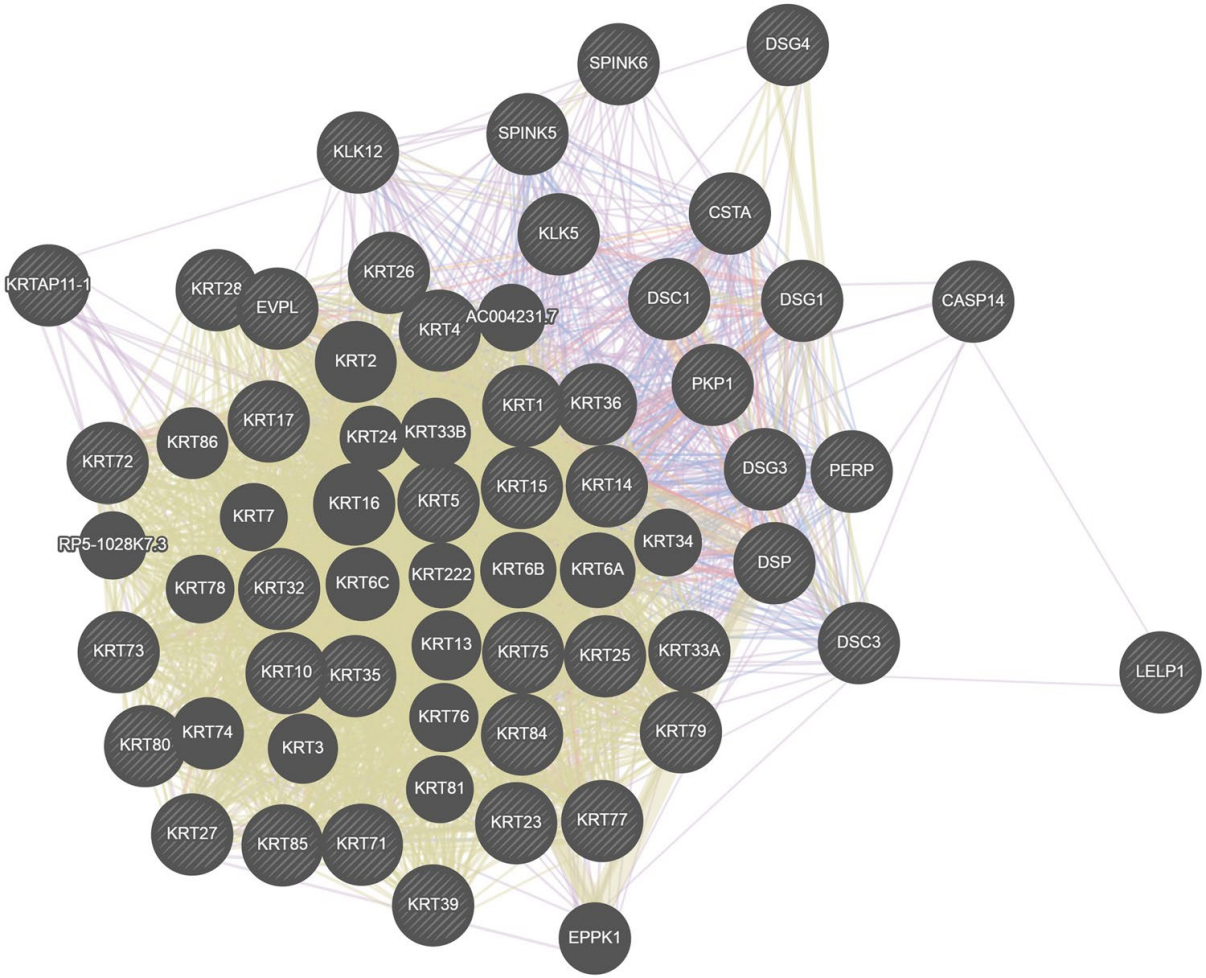
Co-expression network of DEGs involved in the keratinization pathway based on GeneMANIA (genemania.org).

Amongst the DEGs, 524 genes with higher expression and 53 genes with lower expression with a fold change >2, were considered for further analysis. For the highly expressed genes, a total of 14 annotation clusters were identified (enrichment score of >0.5 and p_adj_ < 0.05). The most enriched clusters in decreasing order of enrichment score were structural molecule activity, cornified envelope, differentiation, serine-type endopeptidase inhibitor activity and cell adhesion. The most prominent clusters for the genes with lower expression were transmembrane helix, immunoglobulin subtype, signal peptide and calcium ion binding. Significant Gene Ontology terms for the genes with higher expression in Changthangi goats included cell differentiation, multicellular organism development, keratinocyte differentiation, hair follicle morphogenesis and establishment of skin barrier. Most of these genes were components of nucleus, cytoplasm, extracellular exosome, intermediate filament, keratin filament and cytoskeleton. Genes with lower expression were integral components of membranes and extracellular matrix and were mainly related to transcription, homophilic cell adhesion via plasma membrane adhesion molecules and cellular response to extracellular stimulus. KEGG enrichment analysis highlighted that the DEGs were associated with pathways involved in *Staphylococcus aureus* infection, estrogen signaling and nicotine addiction. As per reactome pathway database, significantly enriched pathways in the dataset included formation of the cornified envelope, keratinization and developmental biology. For this study, we focused on the keratinization pathway since a substantial proportion of DEGs (43 genes) showed higher transcript abundance in Changthangi goats for this process (Table 2). A co-expression network constructed for these 43 DEGs is depicted in Fig. 2 and details of the function of genes in the network are shown in Table 3.

**Table 2:** Expression level of DEGs involved in the keratinization pathway in Changthangi goats.

S.No	Gene	Gene name	Fold change (Positive)
1	KRT39	Keratin 39	5.67
2	LELP1	Late cornified envelope like proline rich 1	5.39
3	KRT33A	Keratin 33A	5.33
4	KRTAP11-1	Keratin associated protein 11-1	5.18
5	KRT25	Keratin 25	5.11
6	SPINK6	Serine peptidase inhibitor, Kazal type 6	5.07
7	KRT27	Keratin 27	5.03
8	KRTAP3-1	Keratin associated protein 3-1	4.99
9	KRT75	Keratin 75	4.97
10	CASP14	Caspase 14	4.84
11	DSG4	Desmoglein 4	4.8
12	KRT23	Keratin 23	4.75
13	KRT71	Keratin 71	4.65
14	CSTA	Cytostatin A	4.63
15	KRT85	Keratin 85	4.61
16	DSC3	Desmocollin 3	4.6
17	KRT17	Keratin 17	4.54
18	KRT32	Keratin 32	4.52
19	KRT73	Keratin 73	4.48
20	DSC1	Desmocollin 1	4.45
21	KRT35	Keratin 35	4.38
22	KRT5	Keratin 5	4.37
23	KRT28	Keratin 28	4.34
24	DSG1	Desmoglein 1	4.32
25	KRT15	Keratin 15	4.3
26	KRT14	Keratin 14	4.26
27	DSP	Desmoplakin	4.23
28	KRT72	Keratin 72	4.05
29	KRT84	Keratin 84	3.83
30	KRT1	Keratin 1	3.75
31	PERP	PERP, TP53 apoptosis effector	3.62
32	KRT36	Keratin 36	3.53
33	KRT10	Keratin 10	3.53
34	KRT77	Keratin 77	3.46
35	KRT79	Keratin 79	3.43
36	DSG3	Desmoglein 3	3.41
37	KRT26	Keratin 26	3.36
38	KLK12	Kallikrein related peptidase 12	3.3
39	SPINK5	Serine peptidase inhibitor, Kazal type 5	2.93
40	KRT80	Keratin 80	2.92
41	EVPL	Envoplakin	2.44
42	KLK5	Kallikrein related peptidase 5	2.29
43	PKP1	Plakophilin 1	2.07

**Table 3:** Number of DEGs in the co-expression network and their function during keratinization.

S.No	Function	FDR	Genes in network	Genes in genome
1	Intermediate filament	1.22E-23	15	42
2	Intermediate filament cytoskeleton	1.63E-20	16	87
3	Skin development	5.23E-19	17	140
4	Epidermis development	5.23E-19	17	141
5	Structural constituent of cytoskeleton	5.54E-11	10	68
6	Keratin filament	2.38E-07	5	10
7	Epidermal cell differentiation	8.01907E-06	7	71
8	Keratinocyte differentiation	3.8602E-05	6	52
9	Intermediate filament cytoskeleton organization	0.00030625	4	16
10	Epithelial cell differentiation	0.00030625	9	263
11	Intermediate filament-based process	0.00030625	4	16
12	Intermediate filament organization	0.006117214	3	10
13	Peptide cross-linking	0.013365359	3	13

Apart from genes for various keratin proteins, expression of genes for some keratin-associated proteins (KRTAPs) was also observed to be much higher in Changthangi goats. Some of the significant KRTAPs were KRTAP7-1, KRTAP11-1 and KRTAP3-1, whose expression was five folds higher.

### Network analysis

**Figure 3:**
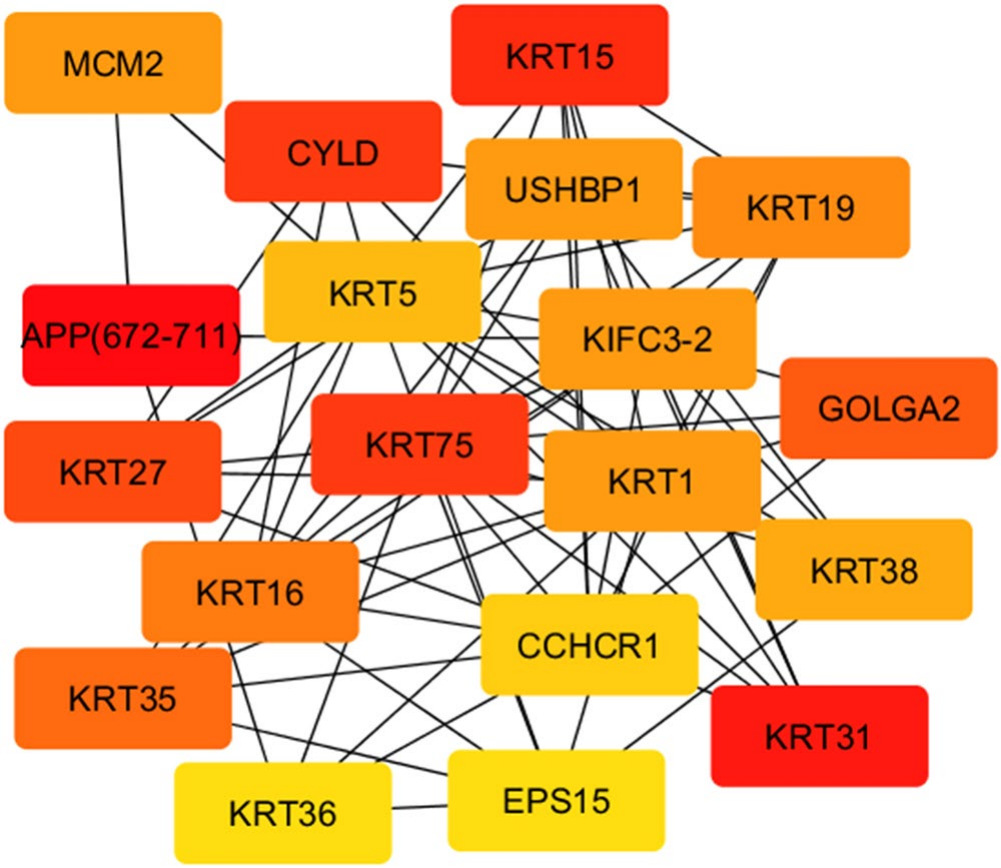
Subnetwork of interactions between the nodes of genes expressed at significantly higher levels in Pashmina producing goats (cytoscape.org).

**Figure 4:**
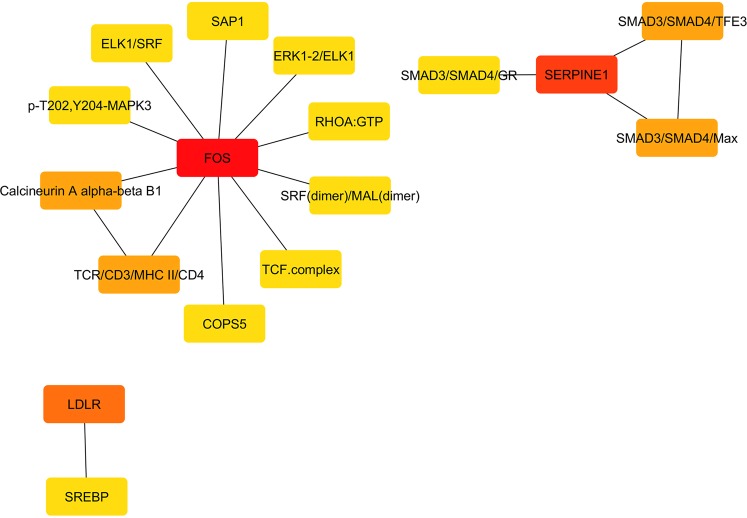
Subnetwork of interactions between the nodes of genes with lower expression in Changthangi goats (cytoscape.org).

Interactions between genes with higher expression (Fold change >2) were analyzed using CPDB and visualized employing Cytoscape ver 3.6.1. Subsequently, a subnetwork was constructed to enrich the interactions between the nodes, with ≥5.0 degree. The top nodes ranked by Maximal Clique Centrality (MCC) scores included genes for keratin proteins (KTR75, KRT5, KRT1, KRT15, KRT16, KRT35, KRT31, KRT27 and KRT38). Similarly, a subnetwork for genes with significantly lower expression identified the most important genes to be FOS, SERPINE1 and LDLR (Figs. 3 and 4).

The top nodes are ranked by Maximal Clique Centrality (MCC) scores and the decrease in score is indicated by change in the color of the node from red to orange.

Another noteworthy observation was that genes such as KRT25, KRT27, KRT17, SOSTDC1 and KRT71 that are involved in hair follicle morphogenesis and DSG4, HOXC13, FOXN1 and DNASE1L2 associated with hair follicle development showed higher transcript abundance in Changthangi goats. In addition, expression of genes linked with the establishment of skin barrier (CLDN1, KRT1, KRT10, GRHL3 and SFN) was also observed to be 2.5 to 5 folds higher in these goats.

### Validation of RNAseq data by qRT-PCR

To validate the results of transcriptomic analysis, five differentially expressed genes were selected at random and subjected to qRT-PCR analysis. The genes that were analyzed were CSTA, FOS, KRT25, MAP28 and PERP. The expression profile of these genes obtained by qRT-PCR showed similar trend with the RNAseq results, thereby substantiating our transcriptome data (Fig. S1).

## Discussion

The present study attempted to investigate global transcriptome profile of skin samples from Changthangi goats that are valued for luxurious fiber called Cashmere/Pashmina in cold desert region of India and Barbari goats that are reared for meat in hot arid regions of the country. Pashmina from Changthangi breed is derived from the secondary hair follicles, whereas hair from non-cashmere goat breeds such as Barbari is obtained from the primary follicles. Analyses for genes with differential expression revealed that the most enriched gene ontology terms were intermediate filament and keratin. There is enough scientific evidence available from different studies suggesting that keratins (KRT) and keratin-associated proteins (KRTAP) are the major structural proteins of hair fiber and sheath. Moreover, their content is considered an important determinant of fleece/wool/hair quality of different species such as humans, sheep, rabbit and goats^5,7–9^. In our study, out of 49 KRT and 30 KRTAP genes annotated in the goat genome, genes for 25 KRT proteins and 3 keratin-associated proteins showed marked up-regulation in Changthangi goats (Fold change >2) (Table 3). Our results are in concert with similar observations in Cashmere goats wherein KRT14, KRT23, KRT25, KRT27, KRT28, KRT80, KRT84, KRTAP3-1 and KRT11-1 were identified to be important for hair follicle morphogenesis in foetal skin at different stages of development^5^. Higher expression of KRT and KRTAP genes such as KRT36, KRT79, KRTAP6-1, KRTAP1-1, KRTAP4-9L, KRTAP9-2 and KRTAP6-2L has also been observed in fine wool Super Merino as compared to coarse wool Small Tail Han sheep^10^. Among these, KRTAPs, KRTAP4-9, KRTAP6-1 and KRTAP6-2L have been reported to determine the physico-chemical properties of the wool fiber and are associated with differences in the crimp of wool^11^. Ovine keratins are the major wool follicle related genes that are expressed in different parts of the follicle. For example, expression of KRT34, KRT38 and KRT39 occurs in cortex, KRT40 and KRT84 in fiber cuticle and KRT25-KRT28 in the inner root sheath^8^. A recent study reported 10 keratin genes to be important candidate genes that regulate hair length in rabbits, which include KRT23, KRT25, KRT26, KRT28, KRT34, KRT38, KRT39, KRT40, KRT7, and KRT84^9^. KRT and KRTAP genes are known to be evolutionarily conserved but their expression trajectory can vary among species due to unique attributes of hair, fiber or wool^12^. Taken together, all these studies suggest that keratins such as KRT23, KRT25, KRT26, KRT27, KRT28, KRT80 and KRT84 can be considered as candidate genes for hair follicle morphogenesis across species.

The skin epidermis represents a major interface between the body and the environment. Keratins are the major intermediate filament proteins of epithelial cells that help to resist mechanical stress and contribute to establishment of skin barrier^13^. We observed >3.5 folds higher expression of KRT1 and KRT10 in Changthangi goats. These keratins are known to be the main structural component of the cytoskeleton in the epidermal outer layer in terrestrial mammals^14^. Other significant candidate genes that are important for the formation of epidermal barrier and showed higher expression in Changthangi goats included the tetraspan transmembrane protein, Claudin 1 (CLDN1), Grainyhead-like 3 (GRHL3) and Stratifin (SFN). CLDN1 has previously been demonstrated to be essential for epidermal differentiation and is an important component of tight junctions in mice^15^. Similarly, GRHL3 is critical for maintaining mammalian epidermal barrier integrity and SFN for epithelial keratinization^16,17^. These observations indicate that Changthangi goats are better equipped to tolerate various mechanical insults as compared to Barbari goats. Marked up-regulation of expression was also observed for genes such as DSG4, EVPL, ACER1, FOXN1, DSP, LELP1, KRT10, CERS3 and CSTA that are associated with keratinocyte differentiation.

A host of transcriptional regulator genes for hair follicle keratin synthesis have been identified in goats, which include GPRC5D, PADI3, HOXC13, FOXN1, LEF1 and DLX3^18^. Some of these transcription factors also emerged as important candidate genes for hair development in the current investigation. In our study, the transcript with highest differential expression between Changthangi and Barbari skin samples was identified to be GPRC5D and its abundance was 8.25 folds higher in Changthangi samples. Previous studies have reported that GPRC5D, a member of RAIG1 family (Retinoic acid-inducible gene-1) is specifically associated with hard keratins. It is expressed in differentiating cells such as the cortical cells of the hair shaft^19^. Higher expression was also evident for PADI3 gene which is involved in differentiation of hair follicles and is known to be expressed in skin epidermis as well as medulla and inner root sheath layers of hair follicles^20^. Human studies have identified HOXC13 as an important transcription factor that regulates the expression of various keratin genes and FOXN1 in nails and hair follicles^21^. In our data also, 5 folds higher expression of HOXC13 was detected in Changthangi goats. Another study in goats reinforced the role of HOXC13 as a regulatory factor governing synthesis of keratin proteins by up-regulating the expression from the promoter of KRT84 and KRT38, whereas down-regulating the expression of KRT1 and KRT2^18^. Interestingly, in our investigation, transcript abundance of FOXN1, KRT1, KRT84 and KRT38 was higher in Changthangi goats. So, the results of the present investigation lend support to the regulatory effects of HOXC13 during hair follicle development. LEF1/TCF3 transcription factor complex is considered important for trans-activating various target genes involved in hair development and cycling^22^. We observed significantly higher expression of these regulatory factors in this study. In fact, LEF1 plays a significant role in development of secondary hair follicles that produce cashmere^23^. Thus, it can be speculated to be involved in cashmere/pashmina production in Changthangi goats. ELF5, an important factor for hair growth and development in humans and mice^4^ also showed almost 7 folds higher expression in Changthangi skin samples. Our results are in concordance with previous studies in different species. For instance, transcriptome profiles of 60- and 120-day-old embryos as well as newborns of Cashmere goats highlighted the importance of GPRC5D, PADI3, HOXC13, PRR9, VSIG8, LRRC15, LHX2, MSX-2 and FOXN1 in hair shaft differentiation and hair follicle keratinization^5^. An attempt to underpin the molecular drivers governing Cashmere hair follicle cycling under different photoperiod conditions (natural and shortened), shortlisted many key regulators including HOXC13, FOXN1 and ELF5 which are essential for the cycling process^4^. The role of HOXC13 in hair shaft differentiation in humans^24^ and FOXN1 in hair morphogenesis in mice^25^ has also been previously substantiated. All these reports suggest that intrinsic molecular mechanisms for development of hair follicles are quite similar in goats, humans and mice.

Wnt signaling pathway has an undisputed role in hair follicle development and hair shaft differentiation^26^. We observed positive regulation of Wnt signaling pathway because of differential expression of genes such as ATP6V0C, ATP6V1C2, HHEX, WNT3, SULF1, CDC73 and LGR6 in our dataset. Relevance of the Wnt pathway in hair follicle differentiation and maturation has also been proven through transcriptome analysis of goat skin at different stages of development^5^. Another study in Inner Mongolia Cashmere goat stressed upon the role of Wnt proteins in regulating dermal papilloma size and hair follicle morphology^27^. In addition, the canonical Wnt pathway is also involved in skin pigmentation and melanogenesis in goats^6^ and chicken^28^.

The microenvironment constituted by the microvascular system amd extracellular matrix (ECM) around the hair follicle is considered important for regulating the structure, metabolism and signaling of dermal papilloma cells (DPCs). These DPCs in turn govern the development, growth and regeneration of hair follicles^27^. Hair follicle development also depends on the communication between cell adhesion molecules and ECM-receptor interactions^5^. The cell adhesion molecules help to relax or reinforce cell contacts in response to increased morphogenetic activity and thus, contribute in moulding the hair follicle^29^. We observed that expression of some genes (SFN, EpCAM, GAPVD1 and PERP) that are involved in cell-cell adhesion was 3–5 folds higher in Changthangi skin. Some of these genes are pivotal for hair follicle morphogenesis. For example, Stratifin (SFN), a regulator of cell cycle is involved in epithelial keratinisation. Owing to its expression exclusively in the keratinocytes, it has been identified as an important signature gene for human DPCs^17^. Mice with mutations in SFN exhibit reduced hair follicle density and repeated epilation (Er) phenotype that is characterized by repeated hair loss and re-growth^30^. The epithelial cell adhesion molecule (EpCAM), a trans-membrane glycoprotein, is expressed in epithelial components of a variety of organs and is involved in cell-cell interactions and maintenance of organ morphology including the hair follicles^31^. Fibrous structural proteins such as collagen, elastin, fibronectin and laminin constitute the ECM. Hair follicle morphogenesis is regulated by ECM-receptor interactions that govern cell proliferation, differentiation and migration^32^. The major genes enriched for ECM-receptor interactions in our goat skin transcriptome analysis included COMP (Collagen oligomeric matrix protein), SV2B (synaptic vesicle glycoprotein 2B), COL11A1 (Collagen 11A1) and SPP1 (Secreted phosphoprotein1) which were highly expressed whereas LAMC3 (Laminin subunit gamma 3) and FN1 (Fibronectin1) were less expressed in Changthangi goats. The expression of various integrins did not vary significantly between the two genetic groups. Comparison of transcriptome profiles of primary and secondary hair follicle derived dermal papilloma cells of cashmere goats revealed differential expression of collagen (CORA1, COEA1), laminin (LAMB3), integrin (ITGA3, ITGA7) and fibronectin genes (FINC)^27^. Similarly, expression of various genes for ECM interaction pathway (ITGA5, ITGA9, COL5A3, COL5A2, COL5A1, THBS2–4) was also reported to vary between embryonic and new born Cashmere goats^5^. Transcriptome analysis of skin of short-hair and long-hair rabbits also witnessed differential expression of COL1A2, COL3A1, COL5A2, COL5A3, LAMA4, LAMC3, ITGB3, TNN and TNXB genes^9^. However, no consistent pattern of expression of genes of the ECM interaction pathway could be observed after analyzing the results of our study as well as other investigations in Cashmere goats and rabbits. Hence, it is reasonable to state that these observations deserve further research in order to pin-point key genes that are pertinent for hair follicle development.

Development and growth maintenance of epithelial appendages including hair depends on a well orchestrated mechanism of cell signaling involving many secretory signals^33^. Of particular interest is Oncostatin M, which is a hair-follicle-expressed factor and an IL-6 family cytokine. It maintains quiescence of hair follicle stem cells and inhibits hair growth by signaling through JAK-STAT5 pathway in mice^34^. Interestingly, we observed that genes for some components of the Oncostatin M signaling pathway such as SERPINE1, FOS and LDLR showed lower expression in Changthangi goats. Thus, it is plausible to speculate that hair growth inhibitory properties of Oncostatin M are less pronounced in Changthangi goats. These observations lend support to ability of these goats to produce much acclaimed cashmere/pashmina fiber. Another noteworthy observation was up-regulation of genes of chemokine signaling pathway (CCL8, CCL26) and an important anti-microbial cathelicidin MAP28 in Barbari goats. Hair-follicle keratinocytes are an important source of chemokine CCL8 that is produced after mechanical stress in skin^35^. Similarly, MAP28 is an important component of the innate immune system of goats which exhibits wide antimicrobial activity against viruses, bacteria and fungi^36^. These observations suggest that cutaneous immunity is better in goats of hot arid region that are more exposed to pathogens as compared to goats of cold desert region.

## Conclusion

In conclusion, the present study offers novel information related to gene networks and metabolic pathways that might play significant role in Pashmina production in Changthangi goats. Our results also identified some candidate genes (KRTs, KRTAPs, GPRC5D, PADI3, HOXC13, FOXN1, LEF1, ELF5, SERPINE1, FOS and LDLR) that can be exploited in future in designing strategies for molecular breeding of Changthangi goats to improve quality and quantity of the finest natural fiber, Pashmina.

## Materials and methods

### Ethics statement

The study was approved by the Institutional Animal Ethics Committee of ICAR-National Bureau of Animal Genetic Resources, Karnal (F.No. NBAGR/IAEC/2017, dated 21.01.2017). All methods were carried out in accordance with guidelines and regulations of the concerned ethics committee.

### Sampling

Barbari skin samples were obtained from ICAR-Central Institute for Research on Goats, Mathura (27.10N, 78.02E and 169.2 m above mean sea level) and Changthangi samples were collected from the breeding tract of these goats in Ladakh (34.10N, 77.34E, 3657.6 m above mean sea level). Four goats of the same age group (15–18 months of age) and sex (bucks) were selected for sampling. Skin samples were collected by a trained veterinarian using biopsy punch under local anesthesia. After aseptically collecting the samples, the tissues were washed with DEPC treated water, finely chopped with surgical blade, transferred to tubes containing RNA later solution and transported to the laboratory. On reaching the laboratory, the RNA later solution was decanted and the samples were stored at −80 °C till further processing.

### RNA extraction and quality analysis

TriReagent (Sigma-Aldrich) was used to extract total RNA from skin samples of four bucks each of Changthangi and Barbari goat breeds. This was followed by on column purification of the isolated RNA using Qiagen RNeasy kit according to the manufacturer’s instructions. RNA concentration and quality were estimated using an Agilent 2100 Bioanalyzer. Only after ensuring that samples have a RIN value greater than 8.0 and OD 260/280 ratio greater than 1.8, they were rendered suitable for RNA-sequencing. Preparation of RNA sequencing libraries was done with Illumina-compatible NEBNext Ultra Directional RNA Library Prep Kit (New England Biolabs, MA, USA). Subsequently, the amplified fragments were sequenced to obtain 2 × 100 bp paired-end reads using Illumina HiSeq. 2500 platform. The raw sequencing data were deposited in the NCBI SRA database under accession number PRJNA62481.

### Mapping of RNA-seq reads to the reference genome

Quality of the raw data generated was assessed using FastQC^37^. For each library, raw reads were pre-processed to remove the adapter sequences, low-quality reads and undermined bases using Cutadapt^38^. All the processed reads were aligned to the *Capra hircus* reference assembly ARS1 using HISAT with the default parameters in order to determine the number of aligned reads and unaligned reads^39^.

### Identification of differential expressed genes and gene enrichment analysis

HTSeq was employed to calculate transcript abundance^40^, followed by analysis of differential gene expression using edgeR^41^. The differentially expressed genes were subjected to functional annotation and enrichment analysis using DAVID^42^ and g:Profiler^43^. Gene Ontology terms with corrected P value less than 0.05 were considered significantly enriched for the differentially expressed genes. Co-expression networks were constructed using GeneMANIA with the network weights reflecting the relevance of each gene in the input list^44^. Construction and visualization of the interaction networks was done using ConsensusPathDB (CPDB)^45^ and Cytoscape ver 3.6.1^46^, respectively.

### Validation of RNA Seq data by quantitative real time PCR

The differential expression of some randomly selected genes namely CSTA, FOS, KRT25, MAP28 and PERP was validated by qRT-PCR. Primer pairs for these genes were designed using Primer 3 software^47^. The details of the primers are given in Table S1. SuperScript III Reverse Transcriptase (ThermoFisher SCIENTIFIC) kit was used to synthesize cDNA for 4 samples each of Barbari and Changthangi breeds using 2 μg of purified total RNA. The qRT-PCR reaction was carried out in triplicate in a final volume of 10 μl consisting of 2 μl of cDNA, 5 μl of SYBR Green Real-Time master mix, 0.3 μl each of forward and reverse primers and 2.4 μl of nuclease-free water) on Roche Light cycler 480 system. GAPDH was used as reference gene to analyze the data by the 2 − ΔΔCT method^48^.

## Supplementary information

Supplementary Information.
