# Supplementary material for: Skin transcriptome profiling of Changthangi goats highlights the relevance of genes involved in Pashmina production
Source: Sci Rep. 2020 Apr 8;10:6050. doi: 10.1038/s41598-020-63023-6 (PMC7142143; doi:10.1038/s41598-020-63023-6)
Supplement: Supplementary file 1 — Supplementary Information. [file 41598_2020_63023_MOESM1_ESM.docx]

**Skin transcriptome profiling of Changthangi goats highlights the relevance of genes involved in Pashmina production**

Sonika Ahlawat^1^, Reena Arora^1^, Rekha Sharma^1^, Upasna Sharma^1^, Mandeep Kaur^1^, Ashish Kumar^1^, Karan Veer Singh^1^, Manoj Kumar Singh^2^ and Ramesh Kumar Vijh^1^

^1^ ICAR-National Bureau of Animal Genetic Resources, Karnal

^2^ICAR-Central Institute for Research on Goats, Mathura

**Table S1. Details of primers used for qRT-PCR analysis**

| **Gene name** | **Primer** | **Sequence (5’-3’)** | **Annealing temperature** |
| --- | --- | --- | --- |
| KRT25 | Forward | GTTCTTGCCGTGGTCTTGAT | 60°C |
|  | Reverse | AGCAGTCAGCCTGGCATTAT |  |
| CSTA | Forward | CTGCCACTCCAGAAATCCAG | 59°C |
|  | Reverse | CTAAAAGCCGGTCAGCTCAT |  |
| PERP | Forward | GCTGCAGTCTAGCGATGACA | 59°C |
|  | Reverse | AGAGGGCAAAGAAGGAGAGG |  |
| FOS | Forward | ACTACCACTCACCGGCAGAC | 60°C |
|  | Reverse | CATTGTAGGTCAGGGCTGGT |  |
| MAP28 | Forward | GCAGTCCTCAGAAGCGAATC | 60°C |
|  | Reverse | CTCTTCGCAGGTGATGTTCA |  |


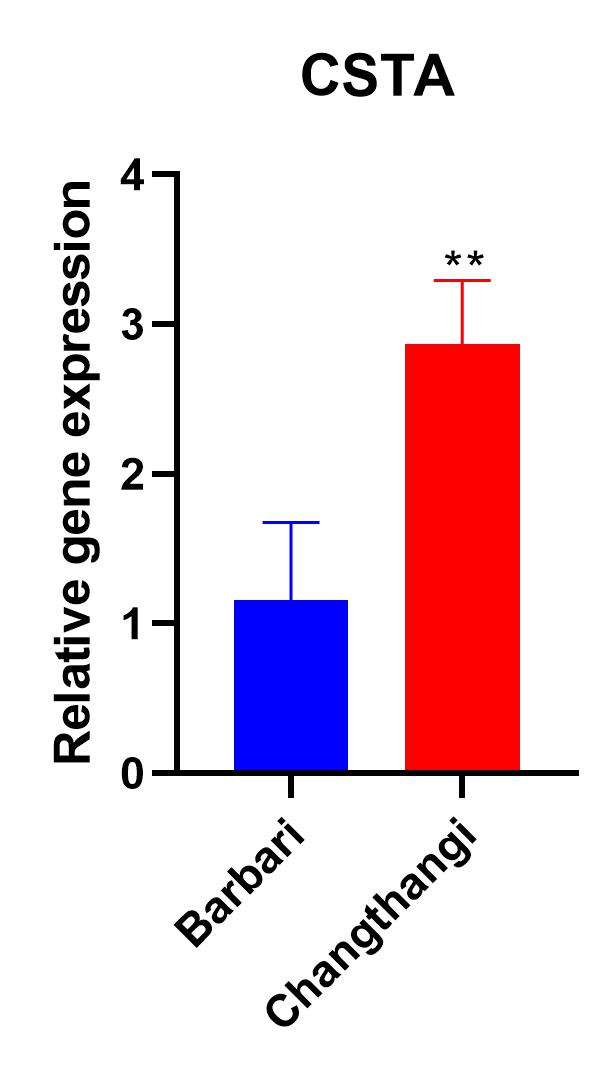

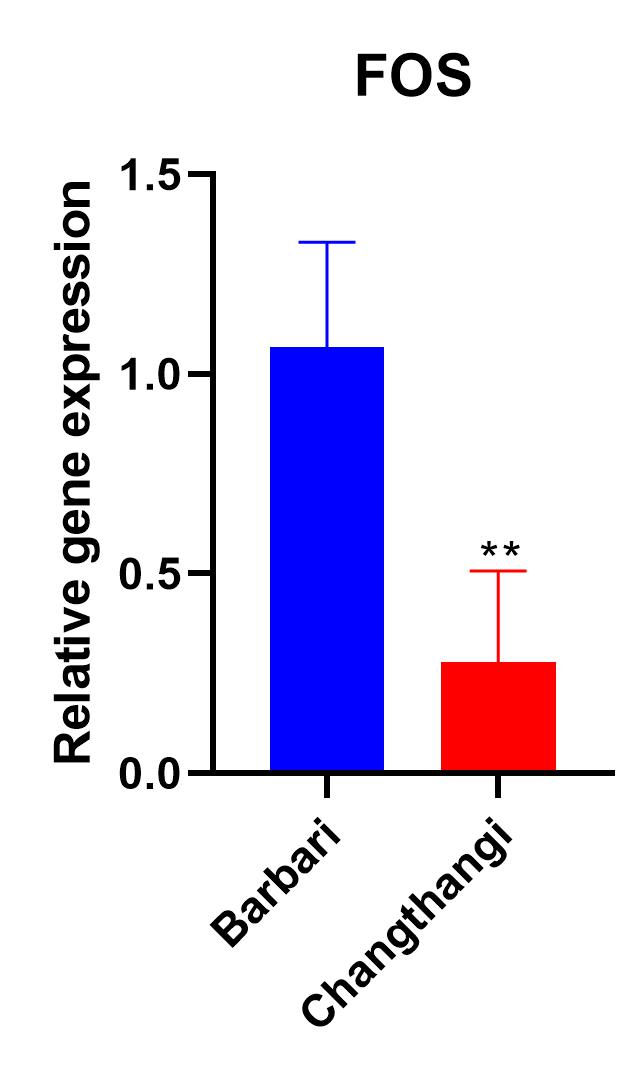


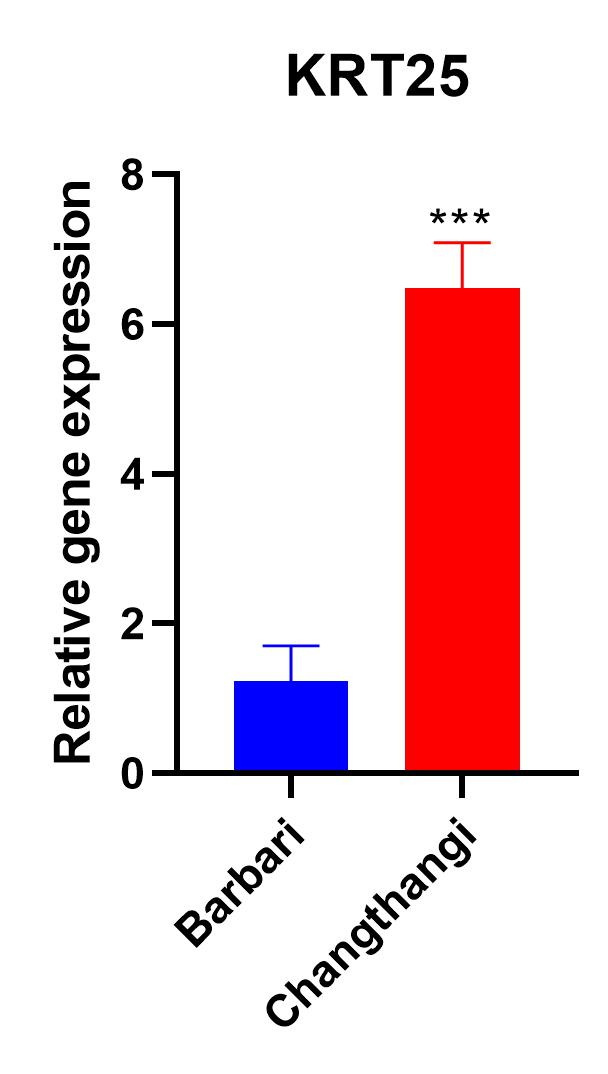

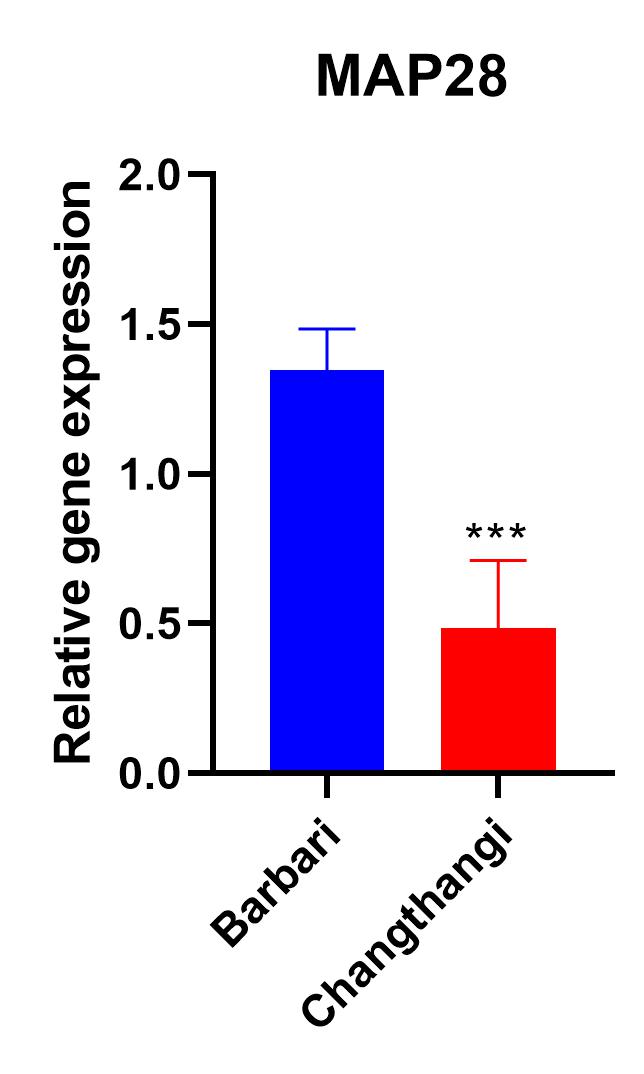


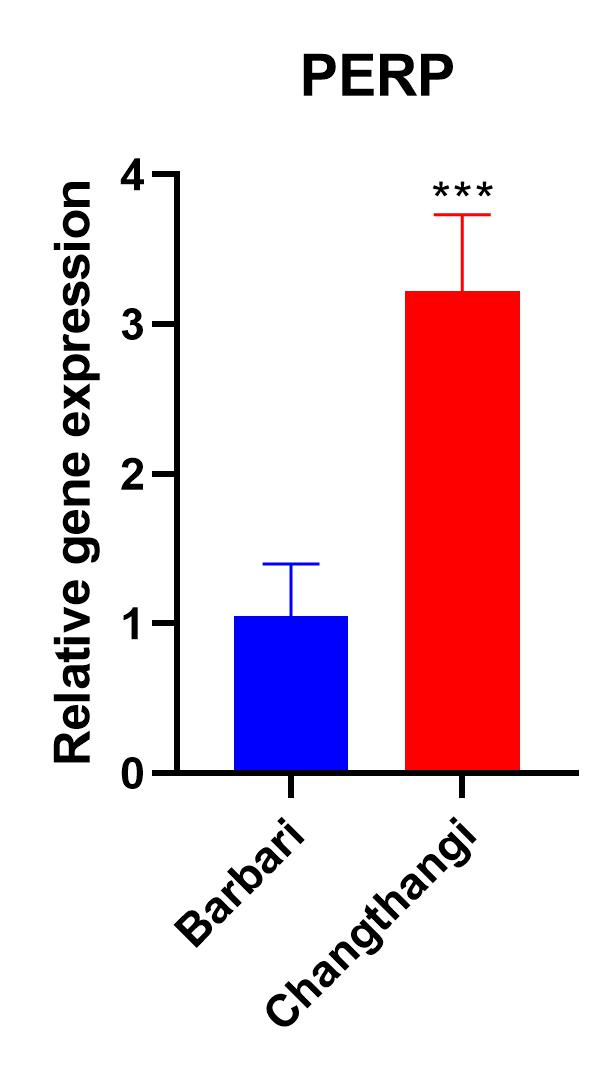


**Figure S1.** Gene expression pattern of differentially expressed genes between Barbari and Changthangi goats by qRT-PCR
